# Three-Dimensional Visualization of Scattered Radiation Sources with a Scintillator-Coupled CMOS Camera in C-Arm-Guided Thoracolumbar Spine Surgery

**DOI:** 10.3390/jimaging12100499

**Published:** 2026-10-10

**Authors:** Toshioh Fujibuchi, Donghee Han

**Affiliations:** 1 Division of Medical Quantum Science, Department of Health Sciences, Faculty of Medical Sciences, Kyushu University, 3-1-1 Maidashi, Higashi-ku, Fukuoka 812-8582, Japan; 2 Department of Radiological Science, Kangwon National University, Samcheok 25949, Republic of Korea

**Keywords:** scattered radiation source, three-dimensional visualization, C-arm fluoroscopy, spine surgery, pinhole collimator, cesium iodide scintillator, CMOS camera, depth camera, occupational exposure, radiation protection equipment

## Abstract

Surgeons performing thoracolumbar spine surgery under mobile C-arm fluoroscopy receive scattered radiation whose spatial distribution cannot be seen. A visualization system combining a pinhole collimator, a cesium iodide scintillator, a high-sensitivity complementary metal-oxide-semiconductor (CMOS) camera and a depth camera was placed at the surgeon’s position, and its images were composed on a three-dimensional point cloud of the operating room. A torso phantom was irradiated once per condition in the lateral (LR) and under-table (AP) projections, without a movable lead-glass plate and with it at two heights. Sources appeared on the beam-entrance surface and the operating table. At the pinhole, 140 cm above the floor, the dose rate without the plate was 29.7 mSv/h (LR) and 15.6 mSv/h (AP), 9.6 and 5.0 µSv per exposure, a ratio of 1.9; a lower edge at 135 cm gave no measurable reduction, and at 120 cm the dose rate fell by 20% (LR) and 6% (AP). Monte Carlo simulation with a validated model of the same tube head gave a ratio of 1.90 at the measurement position and reproduced the null at 135 cm. Projection choice mattered more than plate position.

## 1. Introduction

Fluoroscopically guided procedures have become routine in orthopedic and spinal surgery. Pedicle screw insertion, vertebral body augmentation and fracture reduction all rely on intraoperative imaging with a mobile C-arm, and the surgeon remains beside the operating table throughout the exposure. Among the staff in the room the surgeon consistently receives the highest dose: in a prospective comparison of lumbosacral fusion with fluoroscopy and with navigation, the surgeon’s dose exceeded that of the assistant, the scrub nurse and the radiological technologist in every measured body region during fluoroscopy [1]. Measurements on anthropomorphic phantoms have shown that the scatter dose to the surgeon depends strongly on the C-arm configuration and on the distance from the patient [2], and multicenter data from percutaneous thoracolumbar pedicle screw fixation give mean doses per procedure of 1.7 µSv to the whole body, 204.7 µSv to the extremities and 30.5 µSv to the eye lens [3]. The dose accumulated by a high-volume spine surgeon is therefore not negligible, and it is closely coupled to the dose delivered to the patient [4]. A recent systematic review of minimally invasive cervical spine surgery concluded that per-procedure doses generally remain within occupational limits, but that the evidence is too sparse to define safe thresholds, so dose-reduction strategies remain the priority [5]; no comparable synthesis is available for thoracolumbar procedures, which use higher exposure factors and generate substantially more scatter.

The lens of the eye is the organ that most often sets the protection requirement. International Commission on Radiological Protection (ICRP) Publication 103 [6] retained an equivalent dose limit of 150 mSv in a year for the lens of the eye. Following the ICRP statement on tissue reactions [7], this was lowered to 20 mSv per year averaged over defined periods of five years, with no single year exceeding 50 mSv. Doses to the lens of orthopedic surgeons using the inverted C-arm technique have been reported to reach 0.8 mSv per month, and leaded eyewear reduced the exposure by about 60% [8]. Similar concerns have been raised for scoliosis surgery, where the surgeon standing on the same side as the X-ray tube received a significantly higher dose than the surgeon on the opposite side [9], and for minimally invasive lumbar fusion, where the annual limit for the unprotected lens of the assistant surgeon would be reached after several hundred procedures [10].

The operating room, however, is not equipped for radiation protection in the way an angiography suite is. Ceiling-suspended shields and table-side curtains are rarely available, the sterile field restricts where a movable shield can be placed, and in many institutions a radiological technologist is not permanently assigned to the operating room, so that the choice of exposure parameters and of protective measures is left to the surgical team. Under these conditions an intuitive understanding of where scattered radiation is produced, and of what a given shield actually achieves, is of practical value.

Scattered radiation is invisible, and its distribution in a room is difficult to estimate from experience alone. One way of making it visible is the pinhole camera principle. Chida et al. used an imaging plate (IP) with a pinhole collimator to identify the sources of scattered radiation received by staff during interventional procedures [11]; Noro et al. applied the same principle to the assessment of the air dose distribution in a vertical plane [12]. Both approaches required either a long exposure or repeated irradiations and an off-line readout step, which prevents the image from being obtained during the procedure. Lee et al. replaced the IP with a cesium iodide (CsI) scintillator and a high-sensitivity complementary metal-oxide-semiconductor (CMOS) camera and obtained scattered radiation source images in real time with an exposure time of 50 ms during general radiography [13], where the output per exposure is far higher than in fluoroscopy. A separate pinhole-camera study identified scattered X-ray sources from patient phantoms and equipment during general radiography, CT and interventional radiology [14].

These methods produce two-dimensional (2D) images of scattered radiation sources. Even when an image is superimposed on a visible-light photograph, a single projection fixes the viewpoint: the scatter is seen as it appears from the camera, and how the source, the shield and the operator are arranged relative to one another cannot be judged. Three-dimensional radiation imaging is not itself new: an integrated system combining a Compton camera with three-dimensional scene reconstruction has been used to localize contamination inside a reactor building [15]. Such systems work at gamma-ray energies, however, and equivalent demonstrations in the diagnostic X-ray range remain scarce. We recently addressed this by adding a depth camera to the system and displaying the scattered radiation source image on a three-dimensional (3D) point cloud of a cardiac angiography suite, so that the scene could be rotated and inspected from any direction; this made it possible to confirm visually that a ceiling-mounted shield placed close to the physician removed most of the scatter [16]. That work was carried out with a fixed biplane angiography system, in a room designed for interventional procedures and equipped with a full set of protective devices.

The orthopedic operating room differs in almost every respect. The C-arm is mobile, the projections used during spinal fusion are the lateral and the under-table anterior–posterior views rather than the oblique cardiac views, the beam is more heavily attenuated by the patient and by the carbon table, and the only shield that can usually be interposed is a small movable lead-glass plate. Scattered radiation in a mobile C-arm room has been studied by Monte Carlo simulation, including the visualization of directional vectors of scattered photons [17]. Phantom experiments have also tested a scattered-radiation protector during C-arm-guided spine surgery [18], but the distribution has not been observed experimentally in three dimensions in the room itself.

The purpose of this study was to visualize scattered radiation sources under conditions simulating thoracolumbar spinal fusion with a mobile C-arm, and to examine how the source distribution and the dose rate at the surgeon’s position depend on the irradiation direction and on the position of a movable protective plate. The detector head, with its 5 cm pinhole-to-scintillator distance, the composition software and the analysis are those of [16]; two things distinguish the work from it. First, the two projections that dominate spinal fusion, the lateral and the under-table views, are compared at the operator position with the image and the dose rate recorded together. Second, the shield available in an operating room is a small free-standing lead-glass plate rather than a ceiling-suspended screen. This is, to our knowledge, the first application of the method in a mobile C-arm orthopedic room and the first direct observation of what such a plate achieves in that setting. The measured distributions are compared with a Monte Carlo model of the same C-arm developed in our earlier work [17].

## 2. Materials and Methods

### 2.1. Scattered Radiation Source Visualization System

**Figure 1:**
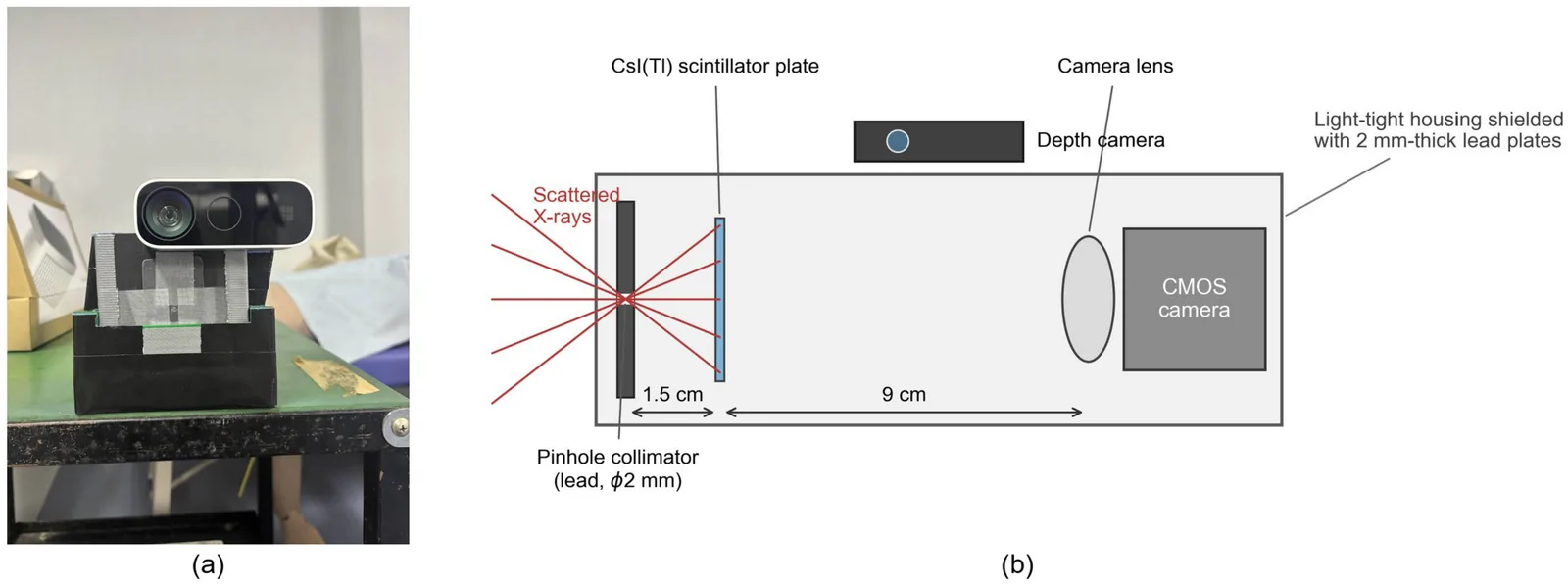
Scattered radiation source visualization system: (**a**) external appearance, with the depth camera mounted on top of the lead-shielded housing; (**b**) schematic of the internal arrangement.

The visualization system used in this study is based on the system described by Lee et al. [13] and extended with a depth camera as in our previous work [16]. It consists of a pinhole collimator, a CsI scintillator plate (J13113; Hamamatsu Photonics K.K., Hamamatsu, Japan), a high-sensitivity CMOS camera (ORCA-Quest; Hamamatsu Photonics K.K., Hamamatsu, Japan) and a depth camera (Azure Kinect; Microsoft Corporation, Redmond, WA, USA). Scattered X-rays that pass through the pinhole are converted to visible light in the CsI scintillator and imaged by the CMOS camera; the resulting distribution is then superimposed on the 3D point cloud acquired with the depth camera. The external appearance and the internal arrangement of the system are shown in Figure 1.

The pinhole collimator was a lead plate 2 mm thick with an aperture 2 mm in diameter. The scintillator was surrounded by 2 mm-thick lead plates so that only radiation entering through the pinhole reached it, and the whole assembly was enclosed in a light-tight housing. The scintillator plate measures 50 mm × 50 mm with an effective detection area of 45 mm × 45 mm, a scintillation layer 400 µm thick and a substrate 0.5 mm thick, as in [16]. The distance from the pinhole to the scintillator was 5 cm, as in [16], and the distance from the scintillator to the camera lens was 9 cm.

The region imaged by the camera at the scintillator plane is about 27 mm in the vertical direction, and wider in the horizontal direction than the 45 mm effective area of the scintillator, which therefore sets the horizontal limit; at 5 cm these dimensions subtend approximately 48° horizontally and 30° vertically at the pinhole. The acceptance of the collimator itself is wider: for a pinhole of diameter d in a plate of thickness t the geometric transmission falls with the angle of incidence and closes at an incidence angle arctan(d/t) from the collimator axis, so that the full acceptance cone is 2 arctan(d/t), which for the present collimator, with d = t = 2 mm, is 90°. The field actually imaged is therefore set by the scintillator and the camera, about 48° × 30°. Within that field the response is not uniform, and it is useful to say by how much. Two effects act together. The response of a pinhole camera falls approximately as cos^4^θ with the off-axis angle θ; and the geometric transmission of a hole of diameter d in a plate of thickness t falls as (2/π)[arccos x − x√(1 − x^2^)] with x = (t/d) tan θ. Their product is 0.88 at 5°, 0.58 at 15° and 0.31 at 24°, the horizontal edge of the field, and the relative response falls to one half at 17.5° from the axis. Brightness at different positions is therefore comparable to within a factor of two within a cone of about 35° full angle about the axis, which contains the full height of the field and the central 70% of its width. No flat-field or angular-response correction was applied. The images are therefore compared between conditions at the same position in the image, using the fixed display window given below, and brightness is not compared between different positions within one image except inside the central cone just defined. The depth camera, whose wide field of view (FOV) is 120° × 120°, was fixed on top of the housing as close to the pinhole as the mechanical arrangement allowed; the residual offset between the two viewpoints introduces a parallax that is quantified in Section 2.1 below and discussed in Section 4.

The geometric blur of a pinhole camera is set by the ratio of the aperture diameter to the pinhole-to-scintillator distance, here 2 mm/50 mm, or about 2.3°; at the pinhole-to-phantom distance of about 1.1 m in this experiment this corresponds to about 4.4 cm on the object, and at the 45 cm distance of the protective plate to about 1.8 cm. The system therefore identifies which structures produce the scatter, and separates a shielded region from an unshielded one, but does not resolve fine detail within a single source.

Images were acquired at a resolution of 1024 × 576 pixels with 4 × 4 binning and a bit depth of 16 bits, as in [16], and were recorded continuously at 2 frames per second with an integration time of 500 ms per frame. The lens had a focal length of 35 mm and was used at f/2.4 (smc PENTAX-DA 35 mm F2.4 AL; Ricoh Imaging Company, Ltd., Tokyo, Japan), the sensor gain was set to High, and the sensor was held at −20 °C by its built-in forced-air cooling. No dark-frame or offset subtraction was applied: with 4 × 4 binning the pixel value with the beam off is about 3200, and the lower limit of the display window used below, 3225, was set just above it. The camera was not synchronized with the X-ray exposure; at 2 frames per second the frame period is 500 ms, so the 1.16 s exposure spanned 2.3 frame periods and its signal was distributed over three consecutive frames, exactly one of which lay wholly within the exposure. The images presented in Section 3 show that fully contained frame, identified in the same way for every condition, so that the images of all six conditions correspond to the same 500 ms of irradiation and may be compared at a fixed display window. The frame-to-frame noise with the beam off was not evaluated, so the width of the display window, 3225–3278, is not expressed here as a multiple of the noise. The pixel values were monitored throughout the experiment to confirm that the detector remained within its linear range and did not saturate.

The scattered radiation source images and the 3D background images were combined with 3D image composition software (rad-point-cloud-mix version 0.1.0; Fume, Iizuka, Japan). The two cameras differ in field of view and in mounting position. The scattered radiation image was scaled within the software until its angular scale matched that of the point cloud, and the residual offset arising from the different mounting positions was removed by translating the center of the FOV of the CMOS camera within the software until the scattered radiation source image coincided with the position of the object that produced it; the two cameras are mounted parallel to each other, so no rotation was required. The translation was determined before the experiment using the procedure described in [16], in which an acrylic phantom half covered with aluminum is irradiated at the pinhole-to-phantom distance used in the measurements, so that the boundary between the aluminum-covered and the uncovered half in the scattered radiation image can be matched to the corresponding edge in the point cloud. The alignment was checked visually on the same edge phantom; the residual misalignment was not quantified, and the way in which it should be reported is discussed in Section 4. Because the correction is a two-dimensional scale and shift rather than a reprojection through the camera extrinsics, it is exact only at the distance at which it was determined. The depth camera was mounted 5 cm above and 3 cm in front of the pinhole collimator, so that the two are separated by a baseline b of 5 cm across the line of sight, and for a shift calibrated at a distance D the residual angular error at a distance q is b(1/q − 1/D); for b = 5 cm and D = 1.1 m this is 3.8° at the 45 cm distance of the protective plate, a displacement of about 3 cm in the plane of the plate, and −1.2° at 2 m. This is larger than the 2.3° geometric blur and different in kind: the blur is a symmetric loss of resolution, whereas the parallax is a systematic displacement, and the reader should bear it in mind when the boundary of a shielded region in the image is compared with the outline of an object in the point cloud. Since the depth camera supplies a depth value for every pixel, the angular map could in principle be reprojected onto the point cloud through the full extrinsic calibration of the two cameras, which would remove the parallax at every distance rather than at one; that is outside the scope of the present work and is identified in Section 4 as the next development of the software. In the composed images the pixel values of the CMOS camera are displayed on a color scale from blue (low) to red (high), and the display window was fixed at 3225–3278 for all conditions so that images obtained under different conditions could be compared directly.

### 2.2. X-Ray Exposure Conditions

Exposures were made with a mobile C-arm system (FUJIFILM DR CALNEO Cross; FUJIFILM Corporation, Tokyo, Japan) in a mock operating room. The current visualization system is not sensitive enough to follow the low dose rate of pulsed fluoroscopy in real time, so the radiography mode was used, which provides a higher output per exposure. The tube voltage and the tube current–time product were set manually and were identical for every exposure, so that the output did not vary between conditions. The beam was not collimated below the size of the image receptor; the corresponding field at the entrance surface of the phantom was approximately 17 × 20 cm in the LR projection and 22 × 27 cm in the AP projection, so that the irradiated volume differed between the two projections by about a factor of 1.8 in area at the entrance surface. This difference is taken up again in Section 3.2, where the ratio between the two projections is reported. The irradiation conditions are listed in Table 1.

### 2.3. Experimental Setup and Protective Plate Configurations

An anthropomorphic torso phantom (Alderson Research Laboratories, Stamford, CT, USA) was placed prone on the operating table in the position used for posterior lumbar spinal fusion, and the lumbar spine was centered in the irradiation field. Two projections were used: the lateral projection with a horizontal beam (LR) and the under-table projection with the tube below the table and the detector above it (AP). The C-arm was brought as close to the phantom as it could be without the X-ray tube contacting the operating table; the resulting focus-to-surface distance was 50 cm for LR and 65.5 cm for AP.

**Figure 2:**
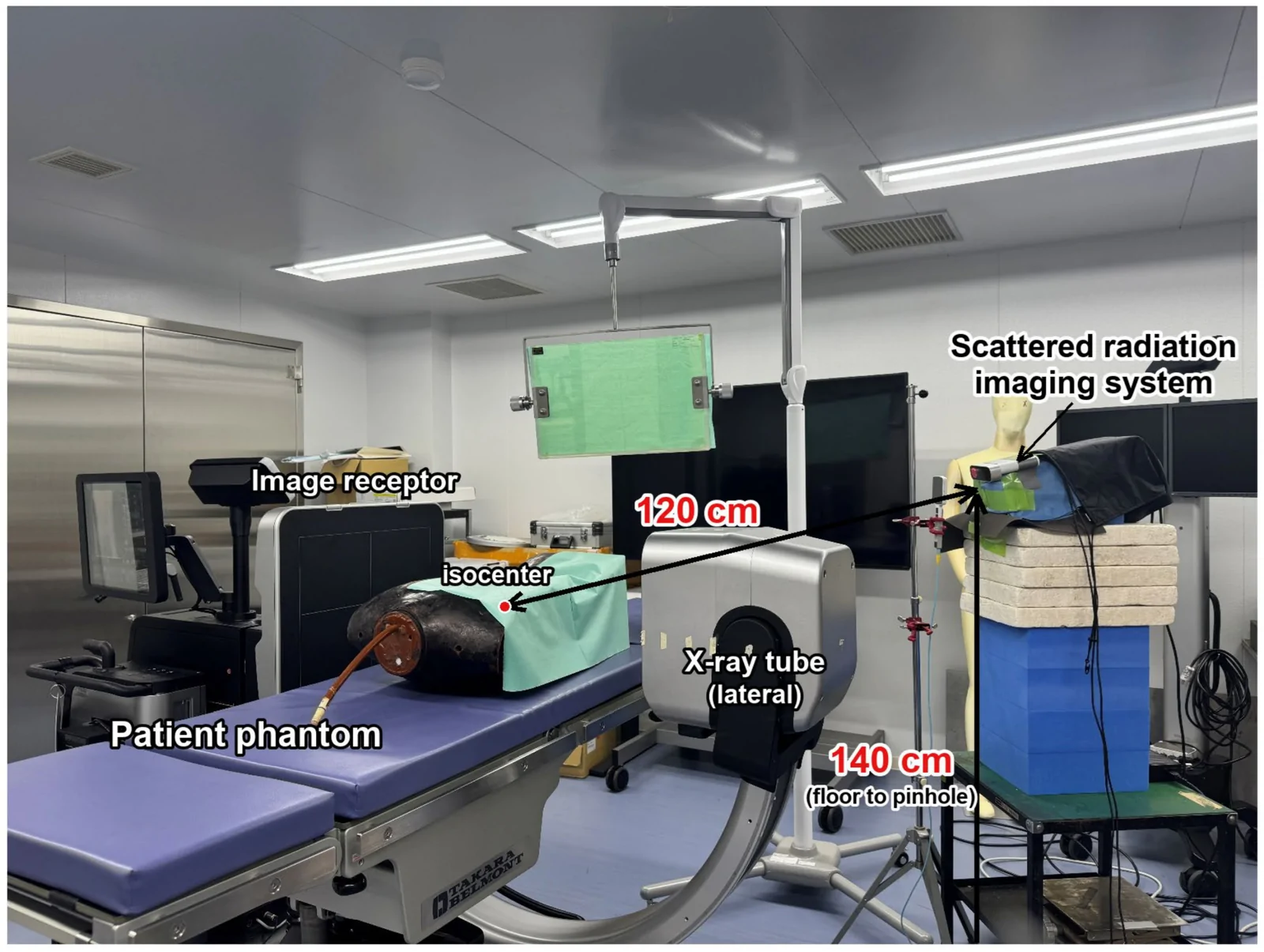
Experimental setup simulating lumbar spinal fusion with a mobile C-arm, in the lateral projection. The scattered radiation source visualization system is placed at the position of the surgeon, with the pinhole 140 cm above the floor and 120 cm from the isocenter; the protective plate, seen here above the table, is shown in its measurement positions in Figure 3.

**Figure 3:**
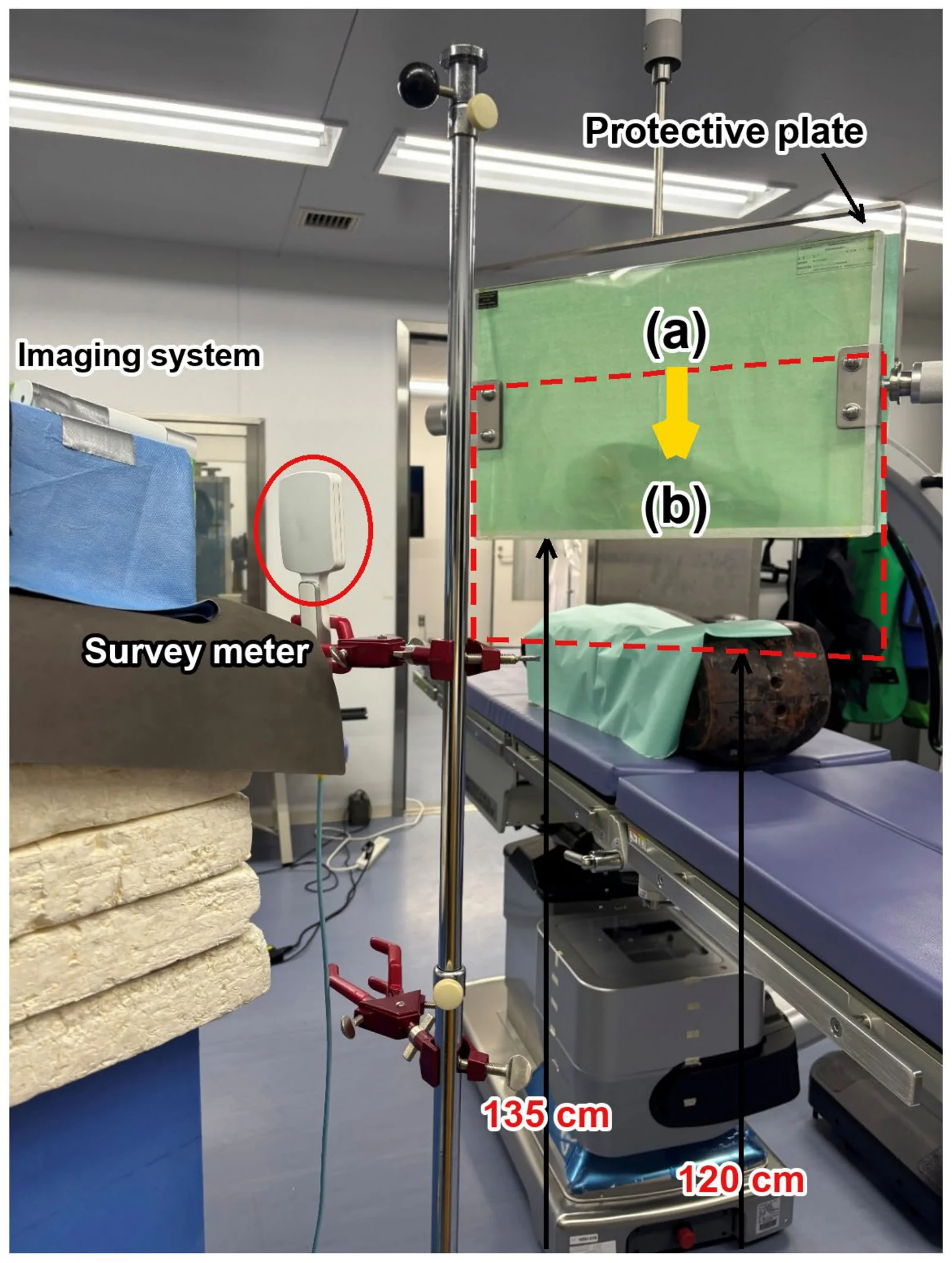
Placement of the movable lead-glass protective plate between the phantom and the visualization system, seen from behind the system with the C-arm in the under-table projection. The lower edge of the plate was set 135 cm (**a**) or 120 cm ((**b**), outline) above the floor; the survey meter was clamped beside the pinhole.

The visualization system was placed beside the table at the position of the operating surgeon, with the pinhole 140 cm above the floor, which corresponds approximately to the height of the chest of a standing adult, and 120 cm from the isocenter. The top of the operating table was 100 cm above the floor. The system stood on the side of the table on which the X-ray tube lay in the LR projection, displaced along the table so that both the phantom and the X-ray tube were within its field of view (Figure 2); seen from above, the line from the isocenter to the pinhole made an angle of 35° with the lateral beam axis. When the protective plate was used it stood 45 cm from the pinhole, between the pinhole and the phantom (Figure 3). These distances are marked in Figure 2 and Figure 3. In the LR projection the system was on the beam-entrance side of the phantom, that is, on the same side as the X-ray tube, because this is the position at which the surgeon receives the highest scatter. The position of the system was kept fixed throughout the experiment so that images acquired under different conditions could be compared.

A movable lead-glass protective plate (ML-II, 0.5 mmPb lead equivalent; Kuraray Trading Co., Ltd., Osaka, Japan) was positioned between the phantom and the visualization system, carried on a floor stand on which the height of its lower edge could be adjusted. The glass panel is 57 cm wide and 37 cm high, so that with its lower edge 135 cm above the floor its upper edge was 172 cm above the floor, and with the lower edge at 120 cm the upper edge was 157 cm; seen from the pinhole, 45 cm away, the panel subtends about 65° horizontally and 42–45° vertically. The panel stood vertically and faced the center of the irradiation field. The height of the lower edge is its height above the floor, and the survey meter described below was clamped beside the pinhole, level with it (Figure 3). Three configurations were compared: without the plate, with the lower edge of the plate 135 cm above the floor, and with the lower edge 120 cm above the floor (Figure 3). Only the height of the plate was varied; its horizontal position, set so that the plate lay between the phantom and the visualization system, was kept unchanged. Because the depth camera cannot image transparent objects, a sheet of paper was attached to the lead glass so that the plate appeared in the point cloud; since the panel has a fixed and known geometry, a clinical implementation would register it as a geometric primitive rather than as measured points, which removes the need for any such marker.

Each combination of irradiation direction and plate position was irradiated once. This single-exposure design is the central limitation of the dosimetric part of the study: no repeat measurement was made, so no standard deviation can be attached to any value in Table 2, and only differences well above the combined reproducibility of the tube output and of the instrument reading can be interpreted. A semiconductor survey meter (RaySafe X2; Unfors RaySafe, Billdal, Sweden) was placed as close to the pinhole as possible, with its sensitive volume at the same height as the pinhole, 140 cm above the floor, and for each exposure the mean ambient dose equivalent rate, H*(10), displayed by the instrument over the exposure was recorded; all dose rates quoted in this paper in mSv/h are this quantity. The manufacturer specifies an uncertainty of 10% for the ambient dose equivalent rate over 20–150 kV (N-series) and a rate range extending to 150 mSv/h, so the values recorded here lie well within the range of the instrument. That figure is a calibration uncertainty and is largely systematic, so most of it cancels when two readings taken with the same instrument at the same position and beam quality are compared; no short-term repeatability is specified, and none was measured with the instrument used. The exposure-to-exposure reproducibility of the output of a radiographic generator is required only to have a coefficient of variation not exceeding 0.05. Taken together these set a floor of a few per cent below which a difference between two single exposures cannot be regarded as real. The purpose of the measurement was to confirm that the intensity of the scattered radiation source in the image and the dose rate at the same position changed in the same direction; the values are used only for relative comparison between conditions, and no correction was applied for the energy or angular response of the instrument.

### 2.4. Comparison with Monte Carlo Simulation

**Figure 4:**
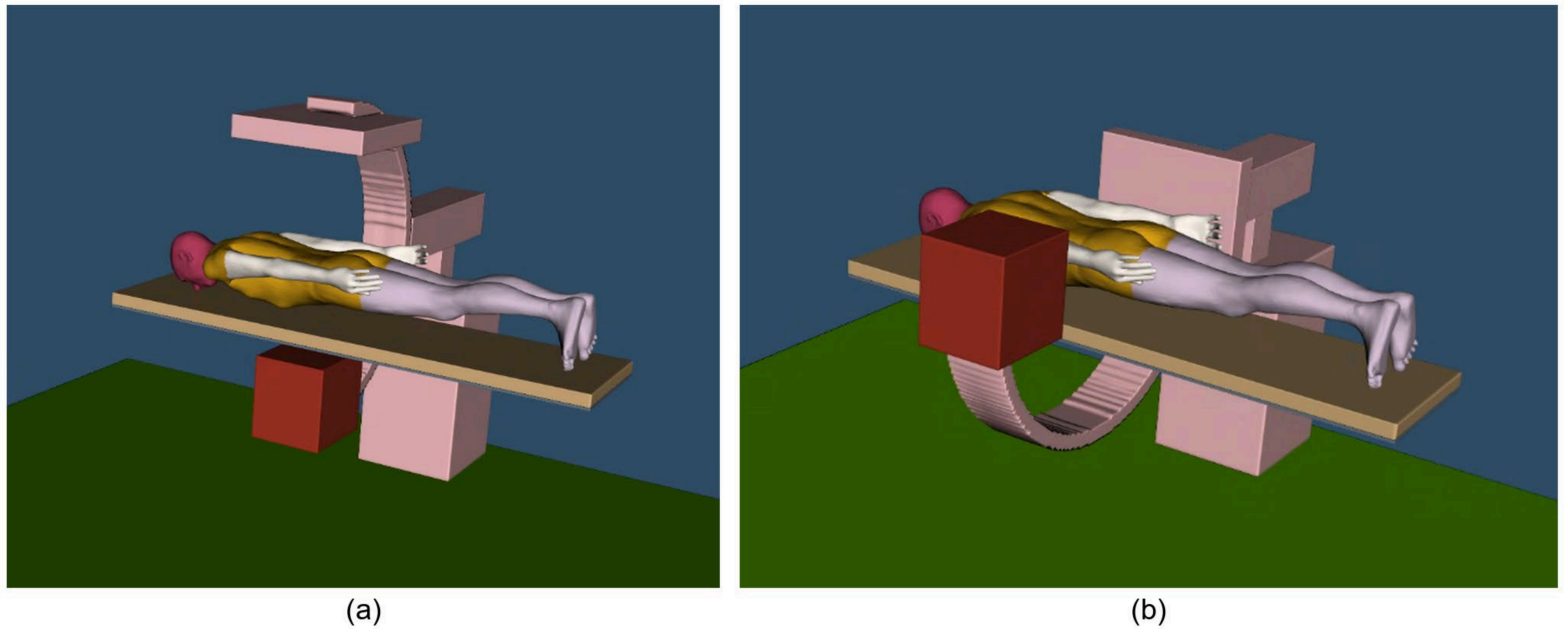
Geometry used in the Monte Carlo simulation: (**a**) under-table anterior–posterior projection; (**b**) lateral projection. The X-ray tube and its housing are shown in dark red; the C-ring, the image receptor and the supporting arm and cart in pink; the operating table in beige; and the floor in green. The colors, including those of the phantom, only distinguish the components and carry no quantitative information.

The measured distributions were compared with a Monte Carlo simulation performed with the Particle and Heavy Ion Transport code System (PHITS) version 3.341 [19]. The C-arm geometry developed in our earlier work on mobile C-arm fluoroscopy [17] was used, which includes the X-ray tube and its housing, the collimator, the image receptor, the C-ring, the supporting arm and cart, and the operating table, together with the ICRP Publication 110 adult male voxel-type reference computational phantom [20]. The phantom was placed prone on the tabletop with the lumbar spine at the center of the field, as in the measurements; unlike the anthropomorphic torso phantom used in the measurements it is a whole-body phantom, so the arms and the legs are present in the simulated geometry and not in the experiment. The source was the tungsten-anode spectrum of [17], with an endpoint of 66 kV, rather than the 80 kV spectrum used in the measurements; the simulation is therefore not matched to the measurement in beam quality, which is one reason why the comparison below is presented as qualitative. The beam was collimated to 35 × 43 cm at the image receptor, with the focus-to-detector distance set to 106 cm and the focus-to-surface distance to 50 cm for the lateral and 65.5 cm for the under-table geometry. The number of histories was 1 × 10^9^. The geometry of the two irradiation directions is shown in Figure 4. The dose distribution on the surface of the phantom and on the operating table, the air dose distribution around the C-arm, and the tracks of the scattered photons were tallied and rendered. This part of the comparison is qualitative: it was used to examine where the scattered radiation is produced and in which directions it travels, and not to reproduce the measured dose rates.

A second set of calculations, performed with PHITS version 3.36, was added in revision to put numbers where the measurement could not reach. The scattering geometry was rebuilt in a form that matches the experiment rather than a reference patient: an elliptical tissue cylinder 30 cm across the table by 20 cm in the anterior–posterior direction and 60 cm long, with a 5 cm cortical-bone spine rod on the beam axis, resting on a 1 cm carbon-fibre tabletop whose upper surface is 100 cm above the floor, in a room with a concrete floor. This stands in for the anthropomorphic torso phantom used in the measurements, whose external dimensions it reproduces and which, unlike a reference computational phantom, has no arms. The 80 kV spectrum was generated by transporting electrons onto a tungsten anode through 2.5 mm Al plus 0.1 mm Cu, giving a fluence-weighted mean energy of 46 keV. The beam was defined by a lead collimator close to the focus; the tube housing and the C-arm structure were not modelled, so the calculated dose rates at the operator position are an upper bound in the lateral projection, where the tube head can stand in the line of sight. H*(10) was scored at the measurement position of Section 2.3, 120 cm from the isocenter at an azimuth of 35°, in a column of 10 cm air cubes, 10 × 10 cm facing the isocenter like the survey meter, from 85 cm to 185 cm above the floor, which gives the values at 140 cm and at 160 cm—the height of the pinhole and the height of the eye lens of a standing adult—and the vertical profile. Each case used 2 × 10^7^ source photons, giving a statistical uncertainty of 1–3% on the scored quantity without the plate. Eight cases were run: the two projections without the plate and with its lower edge at 135 cm and at 120 cm, the under-table projection repeated with the focus-to-surface distance set to the lateral value of 50 cm so that beam direction is separated from focus-to-surface distance, tabletop attenuation and irradiated volume, and the same projection with the tabletop replaced by air. Six further cases covered a field collimated to 20 × 20 cm at the receptor and torso cross-sections of 25 × 17 cm and 36 × 24 cm in place of the 30 × 20 cm reference, addressing the influence of collimation and of patient size that the measurements could not explore. The narrow-beam transmission of the modelled tabletop at this beam quality was calculated separately.

A third set of calculations, also performed with PHITS version 3.36, replaced the collimator of the simplified model by the tube head of the X-ray system used in the experiment, taken from a model of the FUJIFILM CALNEO CROSS whose internal structure was reconstructed from engineering drawings and whose calculated scattered air kerma around the tube head and in the room agreed with measurement within 20% [21]: the 35 cm polycarbonate housing, the iron oil case with its insulating oil, the glass envelope and the 2.5 mm lead cylinder of the insert, the lead scatter shield and the 3 mm lead collimator blades. A flat-panel receptor backed by 1 mm of lead and a hollow aluminium C-arc completed the gantry, and the focal spot was placed at the focus-to-surface distances of the experiment. In the model of [21] the anode–cathode axis lies in the plane of the C, with the anode on the side away from the cart; the cart stood behind the image receptor (Figure 2), so the anode points towards the operator in the under-table projection and upwards in the lateral projection, after the 90° orbital rotation that brings the tube to the operator’s side. The focal spot emitted the 80 kV spectrum into the collimated field, and absolute values per exposure were obtained from the output of the electron-transport calculation of that spectrum, 43.6 µGy per mAs free in air at 1 m. The pinhole was 120 cm from the isocenter and 140 cm above the floor, that is, 117 cm horizontally from the vertical through the isocenter, at an azimuth φ = 35° about that vertical, measured from the lateral beam axis (φ = 0°, straight out from the side of the table) towards the end of the table (Section 2.3). To show how much the comparison depends on that position, H*(10) was scored in air volumes 10 cm wide and 10 cm high, the size of the survey meter, and 5 cm deep, facing the isocenter at 120 cm from the isocenter at φ = 0°, 15°, 25° to 45° in steps of 5°, 60° and 75°, at 140 cm and at 160 cm, and in horizontal maps at both heights. The plate was modelled as a vertical 0.5 mm lead sheet of 57 × 37 cm, 45 cm in front of the pinhole on the line to the isocenter at φ = 35° and facing the isocenter, with its lower edge at 120, 125, 130 and 135 cm; one further case placed it at φ = 0° with the lower edge at 135 cm. Five variants test the assumptions on which the result could depend: the tube head made transparent, which separates its shadow from the rest of the geometry; the under-table projection with its focus-to-surface distance set to the lateral value; the field divided into 64 cones weighted by the cathode–anode dose profile measured on the same system at 80 kV [21], which adds the heel effect; the heel-effect source with the anode orientation reversed; and the polycarbonate exit face of the housing replaced by an acrylic exit window of 15 × 15 cm. To show how much the comparison depends on the height of the survey meter, the cases without the plate and with it at φ = 35° and at φ = 0° were repeated with the scoring volumes centred 135 cm and 145 cm above the floor, 5 cm below and above the pinhole. The lateral cases without the plate used 4 × 10^7^ source photons, those with the plate 2 × 10^7^ and the under-table cases 2.4 × 10^7^; at the measurement point without the plate the statistical uncertainty was 1.1% in the lateral and 2.0% in the under-table projection. The PHITS input decks and the analysis scripts for the calculations described in this section are provided in Supplementary Materials.

Use of Generative AI: Claude (May 2026 version; Anthropic, San Francisco, CA, USA) was used in preparing the submitted manuscript for consistency checking, language and clarity improvement, and assistance with the preparation of the graphical abstract. In the revision, Claude Opus 5.5 (Anthropic) was used to draft revised text and the responses to the reviewers and to write the PHITS input decks and the analysis scripts for the calculations in Section 2.4 and Section 3.3, from the geometry, materials and source data specified by the authors. All outputs were reviewed and revised by the authors.

## 3. Results

### 3.1. Three-Dimensional Visualization of the Scattered Radiation Source

**Figure 5:**
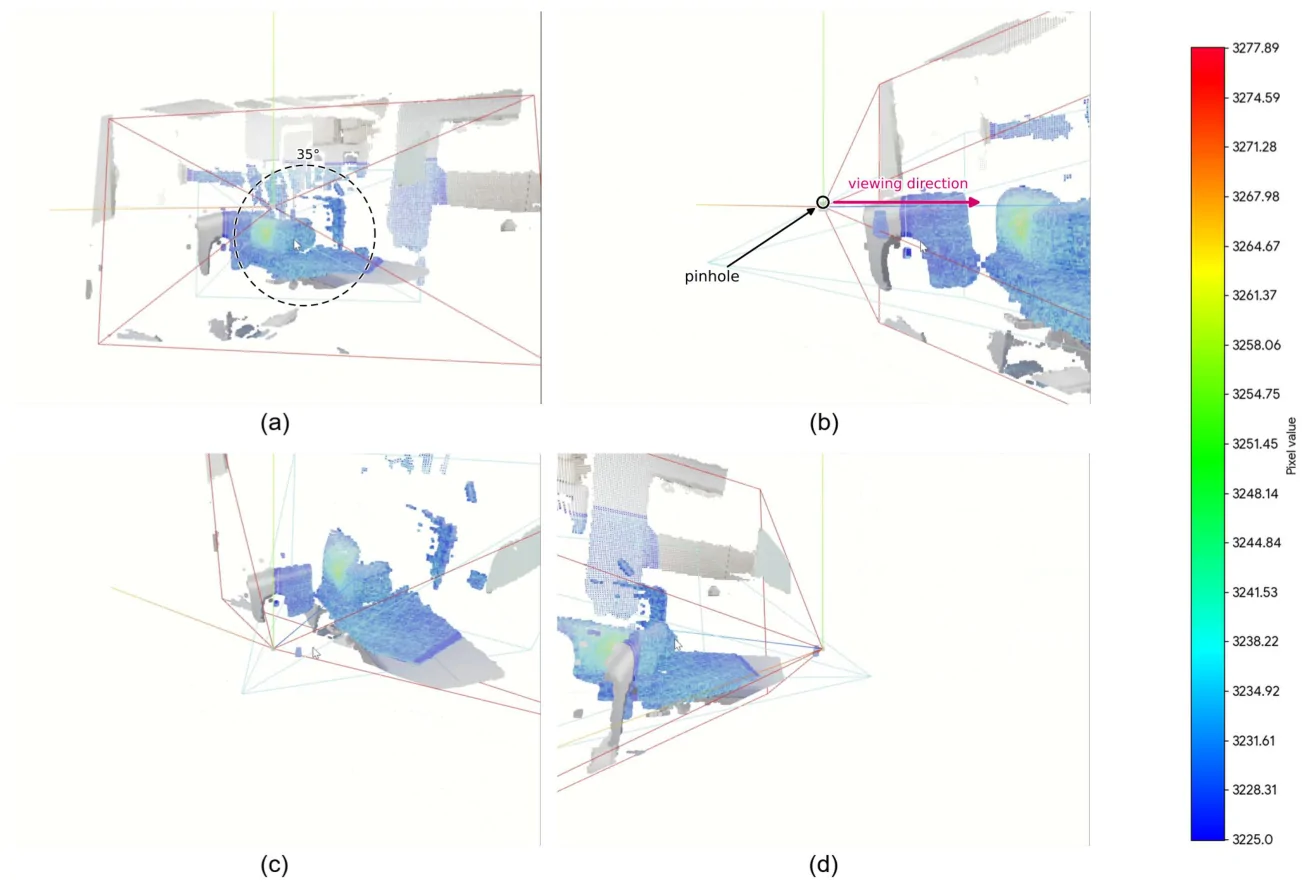
Three-dimensional visualization of the scattered radiation source in the lateral projection without the protective plate, seen from four directions obtained by rotating the point cloud (**a**–**d**). The same scene is shown in every panel. The color scale corresponds to the pixel values of the CMOS camera, with blue indicating low and red indicating high values. The red and light-blue pyramids are the fields of view of the depth camera and of the CMOS camera. In (**b**) the circle marks the pinhole, at the apex of the green axis line, and the arrow the viewing direction, in which the pyramids open. In (**a**), seen along the axis, the dashed ellipse marks where the cone of 35° full angle about the axis of the CMOS camera, within which brightness at different positions is comparable to within a factor of two (Section 2.1), meets the end face of the light-blue pyramid.

Figure 5 shows the scattered radiation source in the LR projection without the protective plate, rendered from four directions. The source appears as a broad region of elevated signal on the surface of the phantom facing the X-ray tube, with signal also present over the operating table. Because no angular-response correction was applied, the relative brightness of features at different positions within one image is not a measure of their relative source strength, and only their locations are read here. Because the background is a point cloud rather than a photograph, the scene could be rotated, magnified and viewed from the head side, from the lateral side and from above, so that the position of the source relative to the phantom and to the surgeon could be read directly from the display.

**Figure 6:**
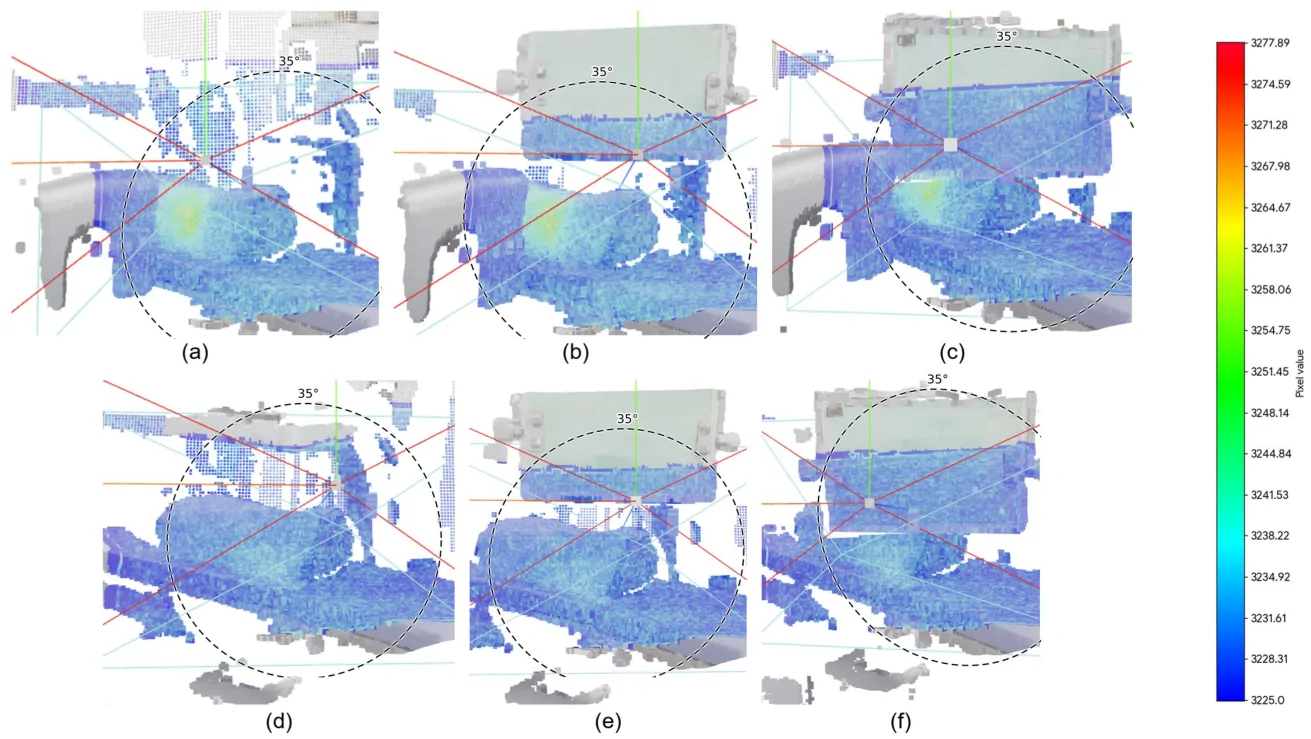
Scattered radiation source images for each irradiation direction and protective plate configuration: (**a**–**c**) lateral projection without the plate, with the lower edge of the plate 135 cm above the floor, and with it 120 cm above the floor; (**d**–**f**) under-table projection under the same three conditions. All six panels use the same color scale for the pixel values of the CMOS camera and the same display window, so that they can be compared directly. The dashed ellipse in each panel marks, as in Figure 5a, where the cone of 35° full angle about the axis of the CMOS camera meets the end face of the light-blue pyramid of its field of view; brightness is comparable to within a factor of two inside it (Section 2.1).

When the protective plate was inserted, the part of the source that lay behind the plate as seen from the system disappeared from the image, whereas the source remained visible above and below the shielded region (Figure 6a,c). Within the angular resolution of the system, the boundary of the shielded region in the image corresponded to the outline of the plate in the point cloud, so the extent of the protection provided by the plate could be judged at a glance.

### 3.2. Effect of the Irradiation Direction and of the Position of the Protective Plate

Figure 6 compares the six combinations of irradiation direction and protective plate configuration. In the LR projection the scattered radiation source was distributed over the entrance surface of the phantom and extended towards the system. In the AP projection the signal recorded at the position of the system was lower, and the region over which scatter was detected lay over the operating table and the caudal part of the phantom. This reflects the fact that the beam enters from below, so that the entrance surface—the surface from which scattered photons escape with the least further attenuation—faces the floor, with the phantom and the tabletop between it and the surgeon.

The dose rates measured at the position of the system are given in Table 2. Without the protective plate the dose rate in the LR projection was 29.7 mSv/h, 1.9 times the value of 15.6 mSv/h obtained in the AP projection; integrated over the 1.16 s exposure these correspond to 9.6 and 5.0 µSv per exposure. Each value is a single reading, so the figures are quoted to three significant figures as displayed by the instrument and no uncertainty can be attached to them; the reasoning below therefore rests only on differences of more than a few per cent. The focus-to-detector distance, the collimation and the tube current–time product were identical in the two projections, so the air kerma–area product of the beam in air, which does not change along the beam axis, was the same despite the different focus-to-surface distances. The entrance field was nevertheless 17 × 20 cm in the LR projection against 22 × 27 cm in the AP projection, so the irradiated volume, and with it the amount of scatter produced, was the larger in the AP projection. In the under-table projection the beam also passes through the carbon-fibre tabletop before it reaches the phantom; the narrow-beam transmission of the tabletop at this beam quality, calculated as described in Section 2.4, is 96.0%, so the reduction in the energy entering the phantom from this cause is small. The thickness traversed, the attenuation path of the scattered photons and the position of the scattering region relative to the measurement point all differ between the two projections as well. This ratio should therefore be read as that between two clinically realizable configurations rather than as the effect of the beam direction alone, and Section 3.3 separates the two by simulation. Setting the lower edge of the plate at 135 cm changed the dose rate by 0.9% in the LR projection and by 1.3% in the AP projection; differences of this size lie within the reproducibility expected of a single exposure and of the survey meter reading, and are therefore treated as no measurable reduction. Lowering the plate to 120 cm produced a clear reduction, 20.2% in the LR projection and 6.4% in the AP projection. The change in the dose rate followed the change in the intensity of the scattered radiation source seen in the images: no difference could be seen between Figure 6a and Figure 6b, or between Figure 6d and Figure 6e, whereas the shielded region is evident in Figure 6c and, less markedly, in Figure 6f.

### 3.3. Monte Carlo Simulation

**Figure 7:**
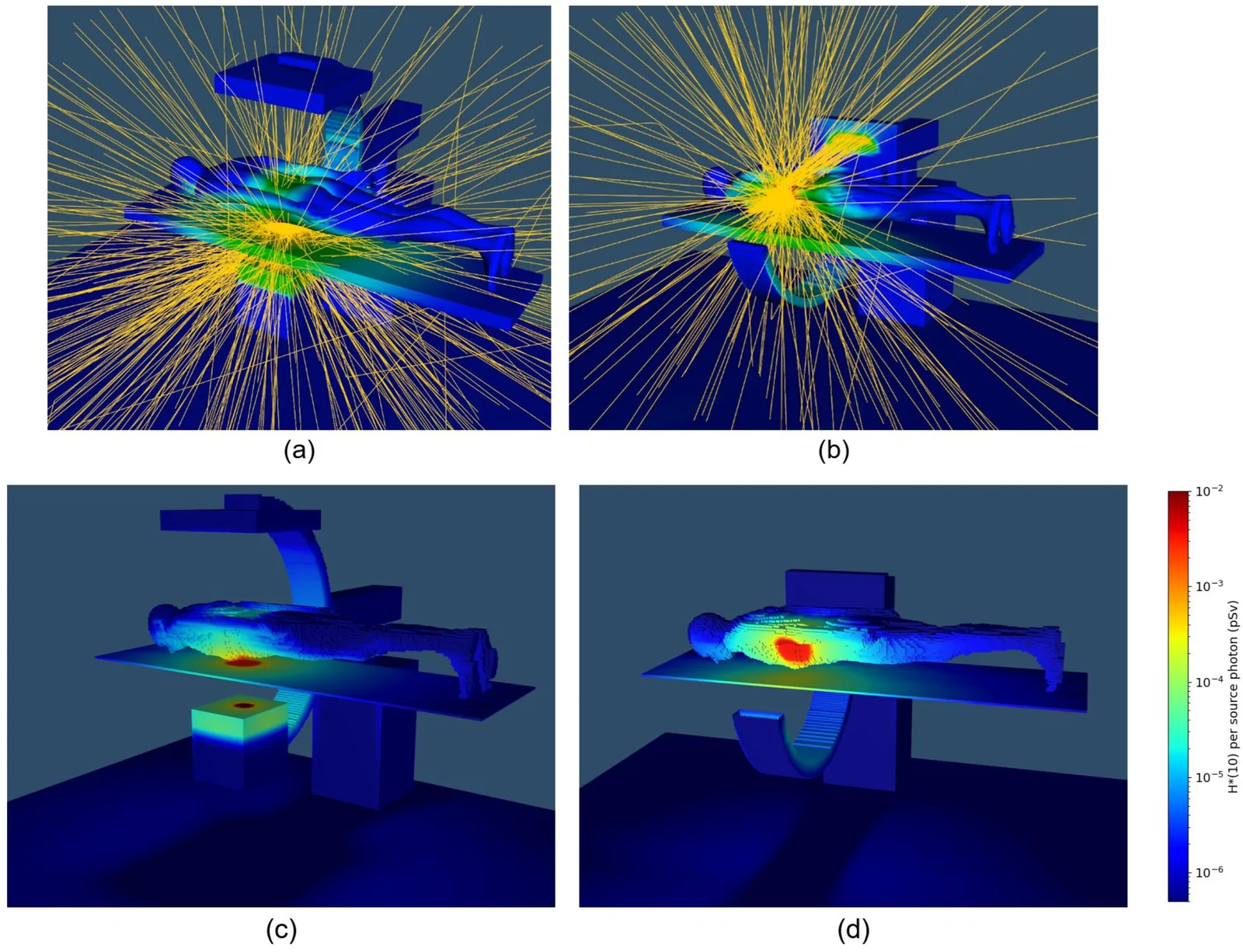
Monte Carlo simulation of the scattered radiation: (**a**) tracks of the scattered photons in the under-table projection; (**b**) tracks of the scattered photons in the lateral projection; (**c**,d) ambient dose equivalent H*(10) per source photon on the surfaces of the phantom, the operating table and the C-arm and on the floor, in the under-table (**c**) and the lateral (**d**) projection, from the same calculation as Figure 8 and on the same logarithmic color scale (color bar); in (**d**) the housing of the X-ray tube, which stands between the viewer and the patient, is left out of the drawing. The surface colors in (**a**,**b**) are qualitative.

The simulated distributions are shown in Figure 7. In the under-table projection the highest surface doses occurred on the anterior surface of the phantom, which faces downwards towards the tube in the prone position, and on the underside of the tabletop where the beam entered, and the tracks of the scattered photons radiated from that region in all directions, with a large fraction directed downwards and towards the X-ray tube (Figure 7a,c). In the lateral projection the hot region lay on the lateral entrance surface of the phantom, and the tracks were concentrated on the tube side of the table, that is, towards the position occupied by the surgeon and by the visualization system (Figure 7b,d).

**Figure 8:**
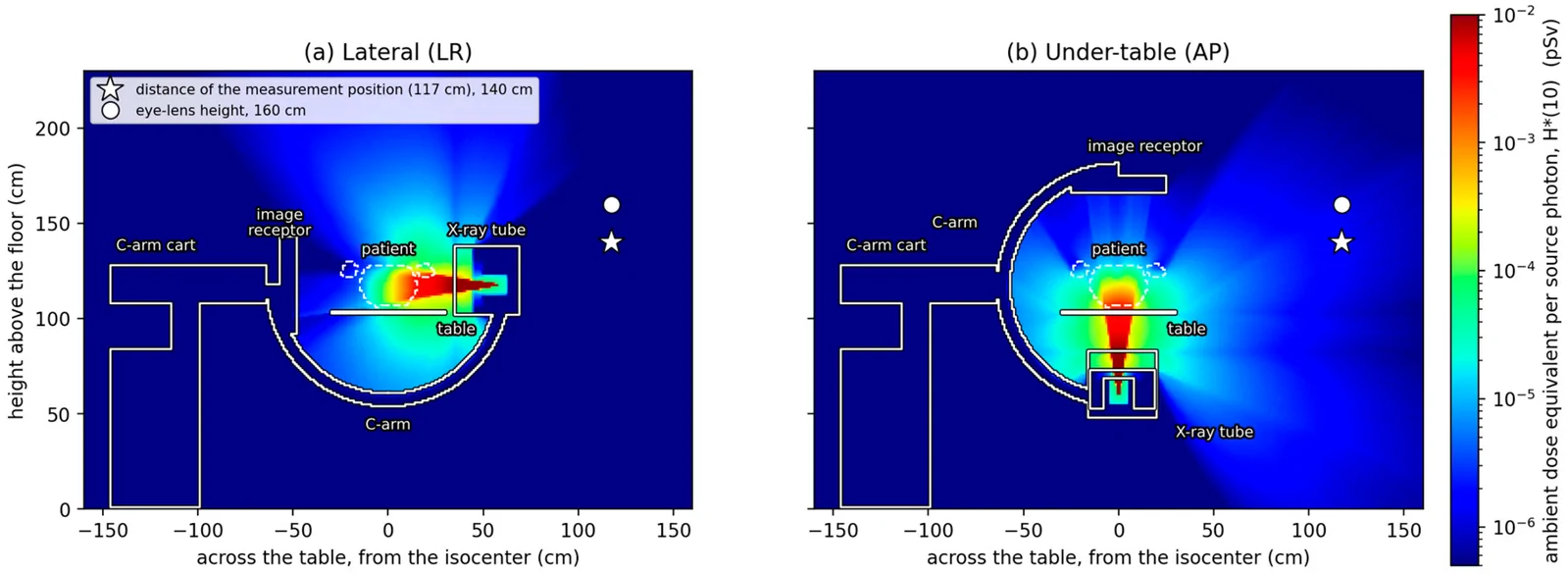
Air dose distribution around the C-arm obtained by Monte Carlo simulation, in the vertical plane through the beam axis: (**a**) lateral projection; (**b**) under-table projection, drawn to scale. The white lines outline the C-arm, the X-ray tube, the image receptor, the cart and the table, and the broken white line the patient, where the plane cuts them; the star and the circle mark the horizontal distance of the measurement position from the isocenter, 117 cm (120 cm from the isocenter at 140 cm above the floor), at the height of the pinhole and at the height of the eye lens of a standing adult; the measurement position itself lay 35° out of this plane (Figure 2). In the lateral projection the elevated region extends back towards the X-ray tube, and in this plane a point at the measurement distance lies in the geometric shadow of the tube housing and the supporting arm, which in this model are treated as solid (Section 3.3).

The air dose distribution around the C-arm (Figure 8) shows the same behavior. The dose is highest immediately adjacent to the entrance surface of the phantom and falls steeply with distance, and the region of elevated dose extends preferentially back towards the X-ray tube. The location of the source is consistent with the measured images. The asymmetry of the air dose distribution is a prediction of the simulation; the present measurement, made at a single point and on visible surfaces only, cannot test it.

**Figure 9:**
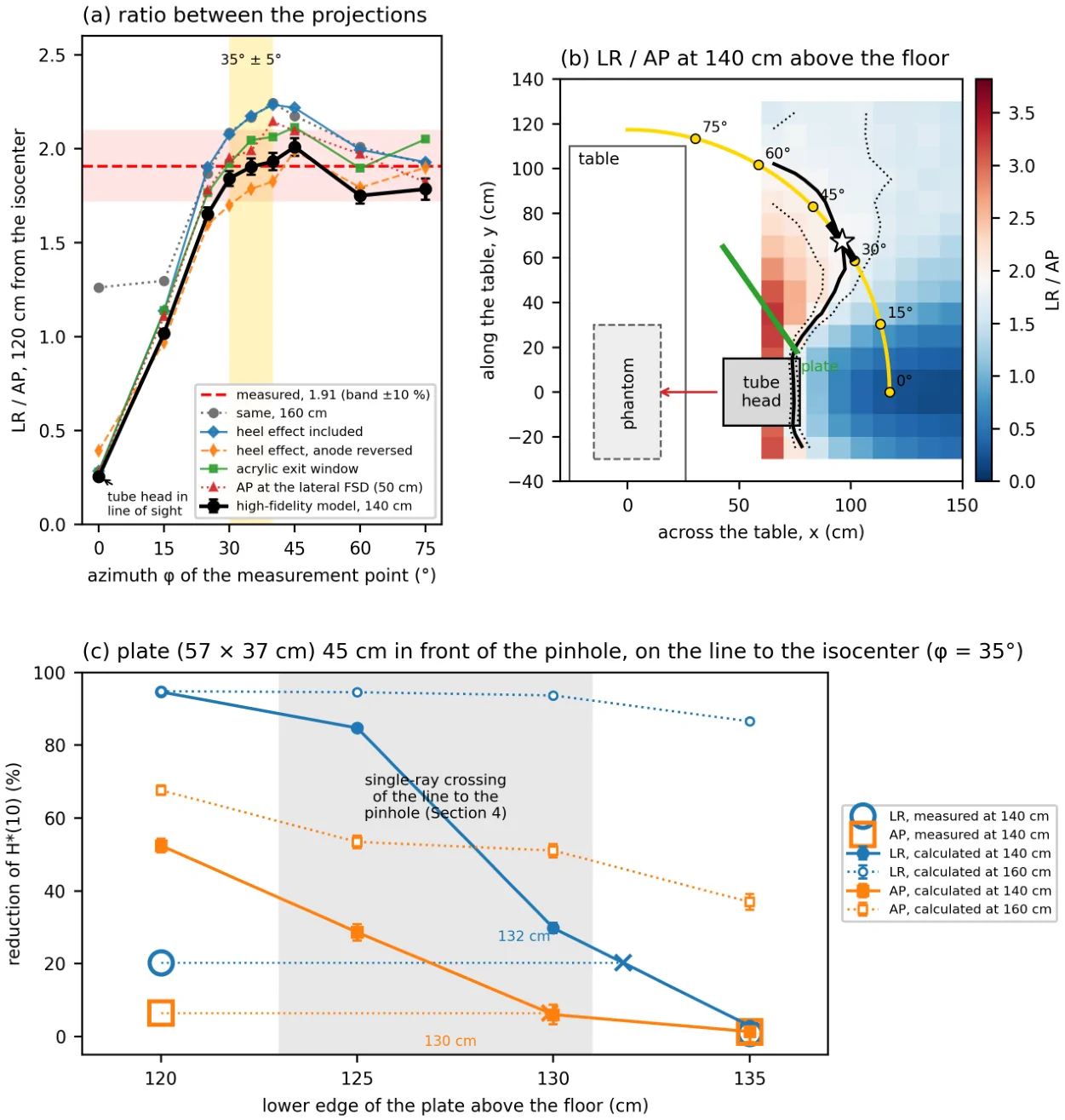
Calculation with the high-fidelity model of the tube head of the X-ray system used in the experiment [21]. (**a**) Ratio of H*(10) in the lateral projection to that in the under-table projection 120 cm from the isocenter and 140 cm above the floor, as a function of the azimuth φ of the measurement point about the vertical through the isocenter (0°, straight out from the side of the table), with the variants described in Section 2.4; the broken line and the pink band mark the measured ratio and ±10%, and the yellow band indicates 5° on either side of the azimuth of the measurement point, which is 35°. (**b**) The same ratio in the horizontal plane 140 cm above the floor, with the arc 120 cm from the isocenter (yellow, with circles at the azimuths at which H*(10) was scored), the measurement position (star, with the thick segment marking 30–40°), the protective plate (green), the outlines of the tube head, the phantom and the table, and the direction of the lateral beam (red arrow); the solid contour marks the measured ratio of 1.91 and the dotted contours ±10%. (**c**) Reduction in H*(10) by the protective plate (57 × 37 cm) placed 45 cm in front of the pinhole on the line to the isocenter at φ = 35°, as a function of the height of its lower edge above the floor, at 140 cm (filled symbols) and at 160 cm (open symbols); the large open symbols are the reductions measured at 140 cm with the lower edge at 120 cm and 135 cm, the crosses mark the heights of the lower edge at which the calculated reduction equals the measured one (about 132 cm in the LR and 130 cm in the AP projection), and the shaded band marks where the single-ray construction of Section 4 places the crossing of the line from the lateral surface of the phantom to the pinhole. Error bars show the statistical uncertainty of the calculation.

The quantitative calculations described in Section 2.4 are collected in Table 3, Table 4 and Table 5 and Figure 9. Scoring H*(10) at the measurement position—120 cm from the isocenter at φ = 35°, 140 cm above the floor—in the model used for Figure 7 and Figure 8 gives 1.52 × 10^−6^ pSv per source photon in the lateral projection and 1.42 × 10^−6^ in the under-table projection, a ratio of 1.07 where the measurement gives 1.9; directly abeam of the tube, at φ = 0°, the ratio is 0.03. In that model the X-ray tube, its housing and the supporting arm are solid blocks of iron, polycarbonate and aluminium that span the whole of the operator’s chest height, so that abeam of the tube the lateral dose falls to the statistical floor of the run (Figure 8), and the model also differs from the experiment in its phantom, a whole-body reference phantom with arms, and in its beam quality. It was built to show where the scattered radiation is produced and in which directions it leaves the patient, which is what Figure 7 and Figure 8 are used for; when used to predict the dose at the operator position it does not reproduce the measurement.

With the gantry omitted altogether, the simplified model of Section 2.4 gives 1.54 × 10^−6^ and 4.64 × 10^−7^ pSv per source photon at the same position, a ratio of 3.32. The two models lie on either side of the measurement, and what separates them is chiefly the gantry, which the first model represents by solid blocks and the second leaves out.

The high-fidelity model supplies the geometry of the tube head, and with it the calculation reproduces the measured ratio (Table 4, Figure 9). In the lateral projection the dose 120 cm from the isocenter depends strongly on the azimuth φ of the measurement point. Straight out from the side of the table (φ = 0°) the line from the beam-entrance surface to a point at chest height passes through the iron oil case of the tube head: H*(10) there is 1.26 µSv per exposure, 19% of its value with the tube head made transparent, and the ratio between the projections is 0.25. As the point moves round the table the line of sight clears the tube head, the lateral value rises and the under-table value falls. At the azimuth of the measurement point, φ = 35°, the ratio is 1.90, against the measured 1.91, and within 5° either side of it the ratio lies between 1.84 and 1.93; the two are equal at φ = 36°. It stays within 10% of the measured value from φ = 30° to 75°, the largest azimuth calculated, and towards the tube it falls to 1.65 at 25° and 1.02 at 15°. No parameter of the calculation was adjusted to the measurement: the distance, the height and the azimuth of the measurement point are those of the experiment. The scatter images agree with that placement: they show the beam-entrance surface of the phantom beside the tube head (Figure 5a and Figure 6a), which the depth camera, mounted beside the pinhole, could not have recorded had the tube head stood in front of it.

At φ = 35° the calculated doses are 7.69 and 4.04 µSv per exposure in the lateral and under-table projections, against the measured 9.6 and 5.0 µSv: both are 80% of the measured value. The two projections fall short by nearly the same factor, which is what an error in the normalization common to both would produce: the tube output was calculated rather than measured, and the survey meter was read without energy or angular correction. A common factor of this kind cancels in the ratio and in the plate reductions, which are the quantities compared here.

The plate provides the independent test, because its effect played no part in locating the measurement point (Table 5, Figure 9c). With the plate 45 cm in front of the pinhole on the line to the isocenter and its lower edge at 135 cm, the calculated reductions at chest height are 2.9% in the lateral and 1.3% in the under-table projection, against the measured 0.9% and 1.3%: the null is reproduced. It would not be at φ = 0°: there the scatter that still reaches the chest in the lateral projection passes just over the tube head, through the region the plate occupies, and the same plate would remove 24.1%, so the measured null is a second indication, independent of the ratio, that the measurement was not made straight out from the side of the table. With the lower edge at 120 cm the calculation gives 94.6% and 52.4%, against the measured 20.2% and 6.4%: the magnitude is not reproduced. Between the two heights the lower edge of the plate crosses the line from the entrance surface to the pinhole, and the calculated lateral reduction falls from 84.8% at 125 cm to 29.8% at 130 cm and 2.9% at 135 cm. The measured 20.2% and 6.4% correspond in the model to a lower edge at about 132 cm and 130 cm respectively, that is, to a plate standing some 10–12 cm higher relative to the line of sight than the nominal 120 cm. The horizontal position of the plate matters as much. A panel of this size removes most of the scatter only while the line of sight passes through it: with the measurement point 10° round the table from the plate the calculated lateral reduction is still 93.0%, but at 25°, where the line from the entrance surface passes just inside an edge of the plate, it is 39.8%, and at 40°, where that line passes clear of the plate, 2.8%; the under-table values at the same two positions are 7.5% and 0.4%. Between these two positions the calculation passes through both measured values. The position of the plate relative to the line of sight was not recorded closely enough to decide between these readings; what the calculation shows is that a few centimetres of plate position decide the outcome.

The details of the model that are least certain change the ratio at φ = 35° by no more than 14%. Adding the heel effect gives 2.18, adding it with the anode orientation reversed 1.79, and replacing the polycarbonate exit face of the housing by an acrylic window 2.05; across the base model and these variants the ratio at φ = 30–40° lies between 1.70 and 2.24. With the focus-to-surface distance of the under-table projection set to the lateral value the ratio at φ = 35° is 1.99, so, as in the simplified model, the difference in focus-to-surface distance works against the measured difference rather than creating it.

The comparison does not depend on the exact height of the survey meter. With the scoring volumes centred 5 cm lower or higher, 135 cm or 145 cm above the floor, the ratio at φ = 35° is 1.81 or 1.91, against 1.90 at 140 cm. The null at 135 cm persists with the meter 5 cm lower, where the plate removes 1.1% in the lateral projection, but not with it 5 cm higher, where it would remove 13.0%; the measured null is therefore consistent with the meter standing level with the pinhole, where Figure 3 shows it, or below it, but not 5 cm above it. With the lower edge at 120 cm the calculated reductions stay between 92.8% and 94.8% in the lateral and between 40.5% and 54.3% in the under-table projection over the three heights, so the height of the meter does not explain why the measured reductions were smaller.

At 160 cm the ratio is 2.17 at φ = 35°, close to its value at chest height, but directly abeam of the tube it is 1.26 rather than 0.25. The top of the tube head is about 130 cm above the floor, so it shields the chest far more than the lens: with the tube head made transparent the dose at φ = 0° rises by a factor of 5.3 at 140 cm and of 1.8 at 160 cm.

The simplified model settles the questions that do not depend on the gantry. Repeating the under-table geometry with the focus-to-surface distance set to the lateral value of 50 cm raises the ratio to 4.41, so the focus-to-surface distance and the larger irradiated volume of the under-table projection act to reduce the ratio rather than to inflate it. Removing the tabletop changes the under-table value by −4.9%: the tabletop adds a little to the scatter reaching the operator rather than removing it, and its narrow-beam transmission at this beam quality is 96.0%, so attenuation of the primary beam in the tabletop is not what separates the two projections. Collimating the field from 35 × 43 cm to 20 × 20 cm at the image receptor reduces the dose rate at the measurement position by 61.3% in the lateral and 81.5% in the under-table projection. Changing the torso cross-section from 30 × 20 cm to 25 × 17 cm and to 36 × 24 cm changes it by −17% and +21% in the lateral projection, so patient size is a first-order influence on the absolute values although it does not affect the comparisons made in this study, which were all made with the same phantom.

The two models built to represent the experiment agree on the vertical distribution, which is the quantity the measurement could not supply. In the simplified model the profile at the measurement position varies by 22% between 100 cm and 180 cm above the floor and the value at 160 cm is 90% of the value at 140 cm in the lateral and 83% in the under-table projection; in the high-fidelity model at φ = 35° the corresponding figures are 95% and 83%. The model of Figure 7 and Figure 8, with its whole-body phantom and solid gantry, gives a steeper fall, 66% and 67%. The unshielded field at an operator position clear of the tube head therefore changes little over the height of a standing adult, and a large difference between chest height and eye height in the shielded case comes from the geometry of the shield rather than from the distribution of the field.

Taken together, the three models establish what the single-exposure experiment could not: that the measured ratio between the projections is reproduced once the tube head of the actual X-ray system is in the model, and is set by where the tube head stands relative to the line from the beam-entrance surface to the operator rather than by the scattering in the patient; that the unshielded field changes little between chest and eye height, so the plate behaves differently at the two heights for geometric reasons; and that collimation reduces the dose at the operator position by more than any position of the plate that was tested.

## 4. Discussion

Scattered radiation sources produced by a mobile C-arm were visualized on a three-dimensional background in a simulated orthopedic operating room. Compared with methods based on an imaging plate [11,12], the combination of a CsI scintillator and a high-sensitivity CMOS camera removes the off-line read-out step, so that the image is available immediately after the exposure rather than after a separate processing stage. With a 1.16 s exposure recorded at 2 frames per second, however, only one frame lies wholly within the exposure, and the present system should be described as producing a single image per exposure rather than as imaging the exposure as it proceeds. Compared with the 2D overlay used in earlier work with this detector [13], the point-cloud background restores the geometry of the room, so that the arrangement of the source, the shield and the observer can be read from any viewpoint rather than only from the one the camera happened to occupy. In practice this is what distinguishes the display from a dose map: the surgeon does not have to translate a number into a mental picture of the room, because the region that produces the scatter is shown on the patient and on the table where it actually is.

It is worth being explicit about what the system measures. The pinhole forms an angular map of the radiation arriving at one point in the room, and the depth camera supplies the geometry of the scene; the display therefore shows the direction from which the radiation reaching the pinhole originates, painted onto the surfaces that the depth camera can see. It does not measure the depth of a source along a line of sight. Where the scatter is produced on a surface the depth camera images—the patient, the table, the C-arm housing—the two coincide and the display can be read as the location of the source, which is the case in all the images presented here. Where the scatter is produced in air, or behind an object the depth camera cannot see, the display places it on the first visible surface along the ray. The same reasoning applies to the residual parallax between the pinhole and the depth camera quantified in Section 2.1: because the correction applied is a two-dimensional scale and shift rather than a reprojection through the camera extrinsics, the mapping is exact only at the distance for which it was calibrated, and a systematic displacement of the order of 3 cm remains at the distance of the protective plate. Since the depth camera returns a depth for every pixel, reprojecting the angular map onto the point cloud through a full extrinsic calibration would remove this term at every distance; that is the next development of the software, and until it is made the residual should be reported as an uncertainty on the position of features in the display rather than treated as a qualitative check.

Registering a measured but invisible quantity onto the real geometry of the operating field, so that it can be read from any viewpoint, is the same operation as an image-guided surgery overlay, and that field has settled a vocabulary for exactly what is at stake here. The residual misalignment discussed in Section 2.1 is a registration error, its value at the structure of interest is a target registration error, and its dependence on the distance between the device and the target is the influence factor that dominates the present arrangement. Reporting the accuracy of a scatter overlay in those terms, with an expanded uncertainty derived from a purpose-made artifact such as the aluminum-and-acrylic edge phantom used here, would place this kind of display on the same footing as other intraoperative overlays and is the form the next validation of the system should take. Both steps have been demonstrated for tracking systems in augmented surgery: a low-cost benchmarking platform qualified the reference chain of a multi-sensor tracking arrangement against an optical coordinate measuring machine and reported the target registration error with an expanded uncertainty [22], and a metrological assessment of augmented-reality tooltip tracking derived accuracy and precision from a purpose-made artifact, with the device-to-target distance treated as an explicit influence factor [23].

In every configuration the images and the dose rates changed in the same direction, or showed no change together. The images were assessed visually and no pixel-value metric was extracted, so the correspondence between pixel value and source intensity is qualitative, and it is a correlation rather than an equivalence. The obvious next step, which requires no new irradiation, is to take the mean pixel value in a fixed region of interest on the entrance surface of the phantom for each of the six conditions and to plot it against the six meter readings; no absolute calibration is needed for that, and six paired points would replace the present verbal statement with a number. That analysis was not carried out for the present paper. The pinhole collimator and the survey meter have different directional responses, so the survey meter also records radiation that does not pass through the pinhole, and the relationship between the two quantities would change with the size and shape of the patient and with the exposure parameters. Quantitative dosimetry was not the aim of this study.

The most practical finding concerns the irradiation direction. The dose rate at the surgeon’s position in the lateral projection was 1.9 times that in the under-table projection. As set out in Section 3.3, a model that includes the tube head of the actual X-ray system reproduces this value at the measurement position, whereas a model of the patient and the table alone gives 3.32, because without the tube head nothing stands between the entrance surface and the surgeon. As noted in Section 3.2 the two configurations differ in focus-to-surface distance and in irradiated volume as well as in beam direction; with the focus-to-surface distance matched the simplified model gives 4.41, so those two factors work against the difference rather than creating it. The images show what dominates it: in the lateral projection the entrance surface of the patient faces the surgeon, so the scatter emerging from that surface travels directly towards them. That surface carries the highest scattered fluence not because the scattering itself strongly favors the backward direction—at 60 keV the Klein–Nishina differential cross section at 180° is about two thirds of its value at 0°, so the distribution is only mildly forward-peaked, and coherent scattering and the incoherent scattering function are set aside here—but because photons scattered near the entrance surface escape with little further attenuation, whereas forward-directed scatter is attenuated by the remaining tissue thickness. Escape attenuation, not the angular distribution of the scattering, is what makes the entrance surface the dominant source. In the under-table projection that high-fluence surface faces the floor, and the phantom and the tabletop lie between it and the staff. The Monte Carlo results reproduce this asymmetry and show that the elevated air dose region extends back towards the X-ray tube. The same directional behavior was reported for the vectors of scattered photons in a mobile C-arm room [17] and underlies the long-standing recommendation to keep the tube under the table. Our results add a visual demonstration of the effect. It follows that, when a lateral projection is unavoidable, standing on the detector side rather than the tube side reduces the dose received; whether this is possible is decided by the surgical approach rather than by dosimetry, so the recommendation is a preference to be exercised where the procedure permits and not a rule. The observation is consistent with the report of McArthur et al. that, during scoliosis surgery, the surgeon standing on the same side as the tube received a significantly higher dose than the surgeon on the far side [9]. The calculation adds a second, less obvious point. On the tube side the dose at chest height depends on where the surgeon stands relative to the tube head: it is lowest directly abeam of the tube, where the tube head itself shields the trunk, and about 6 times higher 35° round the table, at the measurement position, where the surgeon has a clear view of the entrance surface. At the height of the eye lens the same move changes the dose by a factor of only 1.4, because the tube head does not reach that high, so the lens is exposed wherever the surgeon stands on that side and the shielding of the trunk by the tube head should not be mistaken for protection of the eye.

The movable protective plate gave little protection at the height at which it was tested. With its lower edge at 135 cm it produced no measurable reduction at the pinhole 140 cm above the floor, and even at 120 cm it reduced the dose rate by only about a fifth in the lateral projection and by less than a tenth in the under-table projection. The reason is visible in the images, and the geometry makes it quantitative. The tabletop was 100 cm above the floor, the pinhole 140 cm above the floor and 120 cm from the isocenter, and the plate stood 45 cm from the pinhole on the line to the isocenter. Taking the scattering region to lie on the lateral surface of the phantom about 15 cm above the tabletop, the line from that region to the pinhole crosses the plane of the plate about 129 cm above the floor. This construction treats the source as a point, whereas it is spread over the whole entrance surface; the assumption is therefore worth testing, and it is robust: moving the assumed source over the whole height of the lateral surface, 100 cm to 120 cm above the floor, moves the crossing point only between 123 cm and 131 cm, so it stays below the 135 cm edge for every plausible source position. A lower edge set at 135 cm is above that crossing point, so the line passes beneath the plate and no reduction is to be expected, which is what was measured; a lower edge at 120 cm lies below it and the line is intercepted. The construction accounts for the null but not for the magnitude of the reduction at 120 cm, which depends on how much of the extended source the plate occludes as seen from the pinhole. The high-fidelity calculation of Section 3.3 reproduces the null at 135 cm in both projections but not the magnitude at 120 cm: a plate of 57 × 37 cm centred on the line from the entrance surface to the survey meter removes 94.6% and 52.4%, against the measured 20.2% and 6.4%. The measured values are what the model gives when the line of sight passes near an edge of the plate (3–40% and 0–8%), or when its lower edge stands about 130–132 cm above the floor instead of 120 cm, so the four fifths of the dose rate that the plate did not remove in the lateral projection indicate that, as seen from the survey meter, the plate only just reached the scattering region. The practical lesson is the same either way: a small movable plate works only when it is placed squarely on the line of sight, and a few centimetres decide whether it does anything at all. The same construction places the crossing point of the line to the lens of the eye, 160 cm above the floor, at about 141 cm, so a plate whose lower edge is at 135 cm would intercept the line to the lens although it does not intercept the line to the chest; the high-fidelity model gives a reduction of 86.6% at 160 cm with the plate at 135 cm, against 2.9% at 140 cm, at the measurement position. That remains a prediction: the dose rate at lens height was not measured. These results show that the plate does not protect the trunk at chest height; the evidence that it protects the eye comes from the geometry and from the simulation, not from measurement. The contrast with the angiography suite is instructive. In an anthropomorphic-phantom study of coronary angiography, Jia et al. found that a ceiling-suspended screen reduced the operator dose more when placed close to the physician (93.5%) than when placed close to the patient phantom (76.8%), with table-side shielding also used [24]. In our earlier angiography-suite study using the same visualization system, dose rates at a physician-proximal screen position were 13.7–28.0% of the unshielded value, corresponding to reductions of 72–86% [16]. These figures come from different setups, but the reductions are all much larger than the 6.4–20.2% measured here with the movable plate. A shield only works when it intercepts the line between the source and the person; in the operating room the sterile field and the movement of the surgical team make it difficult to satisfy that condition with a movable plate.

The present system provides a direct way of confirming whether a given placement is effective: an anthropomorphic phantom is irradiated once, outside clinical time, in the C-arm geometry planned for the case, and the resulting image shows immediately whether the shield covers the line between the source and the operator, at no additional exposure to the patient or to the team. That is the clinical use we would argue for first, because it needs no change to the operating room and no change to the procedure. The practical consequence of the present results is that, in fluoroscopically guided spine surgery, a movable lead-glass plate of this size, at the distance and the heights tested here, cannot be relied on as the principal protective measure at trunk height. Personal protective equipment—a protective apron, a thyroid collar and leaded eyewear—remains the mainstay, together with optimization of the exposure parameters and of the number of exposures. Collimation deserves particular emphasis: the simulation of Section 3.3 shows that reducing the field from the full receptor size to 20 × 20 cm lowers the dose rate at the operator position by 61.3%, more than the plate achieved in any configuration tested. This is consistent with the report that leaded eyewear reduced the exposure of the eye lens of orthopedic surgeons by about 60% [8], with the emphasis placed on eye protection in percutaneous pedicle screw fixation [3], and with the halving of the surgeon’s dose by collimation in endoscopic spine surgery [25]. Where a movable plate is used, it should stand squarely on the line from the entrance surface to the operator, with its lower edge below the point at which the line to the chest crosses its plane and its upper edge above the point at which the line to the eyes does—about 129 cm and 141 cm above the floor in the present geometry, which the 37 cm panel satisfied with its lower edge at 120 cm—and it should stand as close to the operator as the sterile field allows rather than close to the patient: the nearer a plate of a given size is to the operator, the larger the fraction of the scatter reaching them that it intercepts. The position effect reported by Jia et al. [24] is consistent with this geometric argument.

The study has three groups of limitations. The first concerns the dosimetry. Each combination of irradiation direction and plate position was irradiated once, so no uncertainty can be attached to the values in Table 2 and only the larger differences can be interpreted: the 20.2% obtained with the plate at 120 cm in the lateral projection is secure, the 6.4% obtained in the under-table projection under the same condition is several times the 0.9–1.3% seen with the plate at 135 cm and is treated here as a small but real reduction, and the changes observed with the plate at 135 cm cannot be interpreted at all. Repeating each condition five times and reporting the mean with its standard deviation is the first thing we would do with further access to the room, and until that is done the dosimetric statements in this paper should be read as the outcome of a single trial of each configuration. The measurements were also made at one height, 140 cm above the floor, and at one position. The lens of the eye, which motivates much of the protection effort described in Section 1, lies some 20 cm higher; the simulation reported in Section 3.3 supplies the vertical profile and the effect of the plate at that height, but the values there are calculated and not measured, and the measured values must not be read as lens doses in either direction. Extending the measurement to lens height, and to the positions of the assistant and of the scrub nurse, is the first extension we intend to make.

The second group concerns the instrument and the display. The registration between the CMOS camera and the depth camera is corrected by a two-dimensional scale and shift rather than by a reprojection through the camera extrinsics, so a systematic displacement remains away from the calibration distance, as quantified in Section 2.1. No flat-field or angular-response correction was applied, so brightness may be compared between conditions at the same position in the image but not between positions within one image, which withholds from the reader any statement about how far the source region extends. No pixel-value metric was extracted from the images at all, so the agreement between image and dose rate remains a visual one. The frame rate of 2 frames per second is adequate for observing the onset and the extent of the scatter but not for following rapid changes in the C-arm angle, and the sensitivity of the present detector is insufficient for pulsed fluoroscopy, which is why the radiography mode was used; the absolute dose rates reported here are consequently not those of fluoroscopy and the beam quality differs, whereas the spatial distribution of the sources is governed by the geometry and is expected to carry over. Closing that gap is the principal technical obstacle to clinical use: the tube current–time product used here corresponds to about 22 mA over the 1.16 s exposure, against an average of a few milliamperes in pulsed fluoroscopy, so an improvement in sensitivity of roughly an order of magnitude—through a larger pinhole at the cost of spatial resolution, a thicker scintillator, or summation over many pulses—would be required to follow a fluoroscopic exposure in real time. Finally, the depth camera cannot capture transparent objects, so the lead-glass plate had to be covered with paper to appear in the point cloud; registering the plate as a geometric primitive would remove that difficulty, and the depth noise of the range camera over the 0.5–2 m working range, onto whose reconstructed surfaces the scatter map is painted, was not characterised.

The third group concerns the scope of the experiment. Only the height of the protective plate was varied, so the finding that the plate is of limited use applies to the horizontal placement tested and does not exclude a larger effect at a different distance from the patient. The X-ray field was fixed at one setting, the full size of the image receptor, whereas a clinical operator would collimate more tightly, so the measured scatter is an upper bound; the simulation in Section 3.3 quantifies what collimation would achieve, but this was not measured. A single torso phantom in a single position was used, and the intensity of the scatter changes with the size and habitus of the patient, as the calculations with different torso cross-sections show. The comparison with the Monte Carlo model of Figure 7 and Figure 8 remains qualitative. The quantitative calculations added in Section 3.3 represent the patient by a tissue cylinder of the external dimensions of the torso phantom rather than by an anthropomorphic model; the horizontal position of the plate relative to the line of sight, which decides the reduction obtained with it, was not recorded to the few centimetres that matter; the focal spot is represented by a photon source, with the heel effect added through the measured dose profile, rather than by the electron-source calculation of [21], which gives more scatter around the tube head; and the calculated absolute values fall short of the measured ones by a factor common to both projections. The calculations therefore support the ratio and the comparisons between configurations rather than the absolute dose rates, and recording the position of the measurement point in all three coordinates is the first thing a repeat of the experiment should do.

Beyond the assessment of protective measures, the ability to show scattered radiation in the room where it occurs may have an educational use, although this has not been tested and is offered here as a hypothesis rather than as a finding: no user was involved at any stage of the present work. Surgeons and operating room staff receive little practical training in radiation protection, and it is plausible that an image in which the source of the scatter is visible on the patient, and in which the effect of moving a shield can be seen immediately, is more persuasive than a table of doses; establishing that would require a study with users and an outcome measure. Simulation-based tools approach the same need from the other direction. Süncksen et al. built a virtual-reality C-arm trainer in which Monte Carlo scatter distributions are rendered around a simulated operating room, and both operating room personnel and surgeons reported that it helped them understand intraoperative exposure [26]. What the present system would add is that the display is a measurement made in the room being taught about, with the actual equipment in its actual position, so the geometry is the real one rather than a model of it. In a randomized trial, an audible real-time dose monitor reduced operator exposure during cardiac catheterization without changing patient dose [27], which supports the view that making exposure salient rather than abstract can change behavior.

## 5. Conclusions

A scattered radiation source visualization system combining a pinhole collimator, a CsI scintillator, a high-sensitivity CMOS camera and a depth camera was used to display scattered radiation sources on a three-dimensional point cloud under conditions simulating thoracolumbar spinal fusion with a mobile C-arm. The source was identified on the beam-entrance surface of the phantom and on the operating table, and the distribution could be examined from any direction.

The dose rate at the position of the surgeon in the lateral projection was 1.9 times that in the under-table projection, from one exposure of each configuration. The two configurations differed in focus-to-surface distance and in irradiated volume as well as in beam direction, so the ratio characterizes two clinically realizable configurations rather than the beam direction alone. A Monte Carlo model that includes the validated tube head of the same X-ray system reproduces the measured ratio, 1.90 at the measurement position, and shows that it is set by the direction in which the scatter emerging from the entrance surface travels and by whether the tube head stands in its way: directly abeam of the tube the calculated ratio at chest height is 0.25, while at the height of the eye lens the tube head gives much less protection. At chest height a movable lead-glass protective plate gave no measurable reduction when its lower edge was 135 cm above the floor and reduced the dose rate by 6.4–20.2% when it was lowered to 120 cm, far less than the reduction reported for a well-positioned ceiling-mounted shield in an angiography suite. The Monte Carlo model reproduces the absence of any effect at 135 cm and shows that the reduction obtained with a small plate turns on a few centimetres of its position relative to the line of sight.

These results indicate that, in the orthopedic operating room, the choice between the two projections had a considerably larger effect on the dose rate at the surgeon’s position than the position of the movable shield, and that the proposed system provides a practical means of verifying, visually and in the room itself, whether a given protective arrangement works. Because the movable plate achieved so little at chest height, collimation and personal protective equipment become correspondingly more important; leaded eyewear in particular has been shown to reduce lens exposure during orthopedic C-arm use by about 60% [8], although no protective equipment was evaluated here, and the dose rate at lens height was calculated rather than measured. Repeating the measurements with several exposures per condition, and at the height of the eye lens, is required before the numerical values given here can be treated as anything more than the outcome of a single trial of each configuration.

## Figures and Tables

**Table 1:** Irradiation conditions of the mobile C-arm system.

Item	Value
X-ray system	FUJIFILM DR CALNEO Cross
Mode	Radiography (manual technique)
Tube voltage (kV)	80
Tube current–time product (mAs)	25
Irradiation time (s)	1.16
Total filtration	2.5 mm Al + 0.1 mm Cu
Focus-to-detector distance (cm)	106
Field size at the image receptor (cm × cm)	35 × 43
Focus-to-surface distance, LR (cm)	50
Focus-to-surface distance, AP (cm)	65.5

LR, lateral (left–right) projection; AP, under-table anterior–posterior projection.

**Table 2:** Ambient dose equivalent rate measured with the survey meter at the position of the visualization system for each irradiation direction and protective plate configuration. One exposure per condition (n = 1).

Protective Plate	LR Projection (mSv/h)	AP Projection (mSv/h)
None	29.7	15.6
Lower edge 135 cm above the floor	29.5	15.4
Lower edge 120 cm above the floor	23.7	14.6

LR, lateral (left–right) projection; AP, under-table anterior–posterior projection. Values are the mean ambient dose equivalent rate, H*(10), over the exposure, read from a RaySafe X2 semiconductor survey meter placed as close as possible to the pinhole of the visualization system and at the same height, 140 cm above the floor; no correction was applied for the energy or the angular response of the instrument. Each value is from a single exposure at 80 kV and 25 mAs, so no standard deviation can be given; integrated over the 1.16 s exposure the six values correspond to 9.6, 9.5 and 7.6 µSv in the LR projection and 5.0, 5.0 and 4.7 µSv in the AP projection.

**Table 3:** Ambient dose equivalent per source photon calculated at the operator position, 120 cm from the isocenter at φ = 35°, at chest height (140 cm above the floor) and at the height of the eye lens (160 cm above the floor), for each simulated configuration of the simplified model of Section 2.4, which omits the C-arm gantry. Each value is from 2 × 10^7^ source photons; the statistical uncertainty is 1–3% without the protective plate and up to about 10% behind it. The geometry is the simplified one described in Section 2.4, so the absolute values are not comparable with the survey meter readings in Table 2, and the shielding values are an upper bound because the patient is the only scattering object in the model.

Configuration	H*(10) at 140 cm (10^−6^ pSv per Source Photon)	H*(10) at 160 cm (10^−6^ pSv per Source Photon)	Change at 140 cm Relative to the Same Projection Without the Plate (%)
Lateral, no plate	1.54	1.38	—
Lateral, plate lower edge 135 cm	1.45	0.141	−5.4
Lateral, plate lower edge 120 cm	0.0707	0.0664	−95.4
Lateral, field collimated to 20 × 20 cm	0.595	0.524	−61.3
Lateral, torso 25 × 17 cm	1.28	1.15	−16.9
Lateral, torso 36 × 24 cm	1.87	1.67	+21.4
Under-table, no plate	0.464	0.387	—
Under-table, plate lower edge 135 cm	0.459	0.13	−1.0
Under-table, plate lower edge 120 cm	0.0968	0.0726	−79.1
Under-table, focus-to-surface distance 50 cm	0.348	0.315	−24.9
Under-table, tabletop replaced by air	0.441	0.361	−4.9
Under-table, field collimated to 20 × 20 cm	0.086	0.0708	−81.5

LR and AP as in Table 2. The torso of the reference geometry is 30 × 20 cm in cross-section; the collimated cases reduce the field from 35 × 43 cm at the image receptor; the plate is 57 × 37 cm, 45 cm in front of the pinhole on the line to the isocenter. Calculations performed with PHITS; see Section 2.4 for the geometry, the source spectrum and the assumptions.

**Table 4:** Ambient dose equivalent per exposure calculated with the high-fidelity model of the tube head (Section 2.4) 120 cm from the isocenter, as a function of the azimuth φ of the measurement point about the vertical through the isocenter, and the ratio between the lateral and the under-table projection at chest height (140 cm) and at the height of the eye lens (160 cm).

Azimuth φ (°)	LR, 140 cm (µSv per Exposure)	AP, 140 cm (µSv per Exposure)	LR/AP, 140 cm	LR/AP, 160 cm	LR, 140 cm, Relative to Transparent Tube Head (%)
0	1.26	4.96	0.25	1.26	19
15	4.78	4.70	1.02	1.30	52
25	7.28	4.41	1.65	1.87	81
30	7.75	4.21	1.84	2.09	89
35	7.69	4.04	1.90	2.17	96
40	7.71	3.99	1.93	2.24	103
45	7.26	3.61	2.01	2.18	106
60	5.27	3.01	1.75	2.01	108
75	3.83	2.15	1.78	1.92	123
Measured (Table 2)	9.6	5.0	1.91	—	—

φ = 0° is straight out from the side of the table and 90° would be along it; the measurement point was at φ = 35° (Section 2.3). Anode orientation as in Figure 2, uniform source; statistical uncertainty 1.0–2.9% on each dose and 2.1–3.3% on each ratio. The last column compares the lateral value with that obtained with the tube head made transparent: below 100% the tube head shields the measurement point, above 100% its own scatter adds to the dose.

**Table 5:** Reduction (%) of the ambient dose equivalent by the protective plate, calculated with the high-fidelity model of the tube head with the plate on the line from the measurement point to the isocenter, at chest height (140 cm) and at the height of the eye lens (160 cm), for several heights of the lower edge of the plate.

Azimuth of the Plate/ of the Point (°)	Lower Edge of the Plate (cm)	LR, 140 cm	AP, 140 cm	LR, 160 cm	AP, 160 cm
35/35	120	94.6	52.4	94.8	67.6
35/35	125	84.8	28.7	94.5	53.5
35/35	130	29.8	6.0	93.7	51.1
35/35	135	2.9	1.3	86.6	36.9
35/45	120	93.0	45.7	94.8	64.8
35/60	120	39.8	7.5	37.6	9.0
35/75	120	2.8	0.4	5.5	0.1
0/0	135	24.1	—	91.0	—
Measured (Table 2)	120	20.2	6.4	—	—
Measured (Table 2)	135	0.9	1.3	—	—

Plate: vertical, 0.5 mm lead, 57 × 37 cm, centred on the radius at the azimuth given for the plate, 45 cm inside the arc of measurement points (120 cm from the isocenter) and facing it; reductions relative to the same projection and point without the plate. Where the two azimuths differ (35/45, 35/60 and 35/75) the plate is increasingly off the line of sight of the point: the line from the entrance surface passes through the plate, just inside its edge and clear of it, respectively. Statistical uncertainty 0.4–4.2% (absolute) on each reduction. φ = 35° is the azimuth of the measurement point; at φ = 0° the tube head already lies in the line of sight in the lateral projection. The measured reductions of 0.9% and 1.3% lie within the reproducibility of a single exposure (Section 3.2).

## Data Availability

The data presented in this study are available on request from the corresponding author. The PHITS input decks and analysis scripts used for the calculations reported in Section 2.4 and Section 3.3 are provided in the Supplementary Materials. The 3D image composition software rad-point-cloud-mix version 0.1.0 was developed in-house and is available from the corresponding author on request.

## Supplementary Materials

The following supporting information can be downloaded at: https://www.mdpi.com/article/10.3390/jimaging12100499/s1, Supplementary Materials: PHITS input decks and analysis scripts for the Monte Carlo calculations in Section 2.4 and Section 3.3 (ZIP archive).

## Abbreviations

The following abbreviations are used in this manuscript:

AP	Anterior–posterior
CMOS	Complementary metal-oxide-semiconductor
CsI	Cesium iodide
FOV	Field of view
ICRP	International Commission on Radiological Protection
IP	Imaging plate
LR	Lateral (left–right)
PHITS	Particle and Heavy Ion Transport code System
2D	Two-dimensional
3D	Three-dimensional
